# Integrated Transcriptomics–Proteomics Analysis Reveals the Response Mechanism of *Morchella sextelata* to *Pseudodiploöspora longispora* Infection

**DOI:** 10.3390/jof10090604

**Published:** 2024-08-26

**Authors:** Shurong Wang, Jingyi Wang, Tengyun Wang, Tonglou Li, Lijing Xu, Yanfen Cheng, Mingchang Chang, Junlong Meng, Ludan Hou

**Affiliations:** 1 College of Food Science and Engineering, Shanxi Agricultural University, Taigu, Jinzhong 030801, China; wzlj2005@163.com (S.W.); wangjingyi0304@163.com (J.W.); wangtengyun2021@163.com (T.W.); 15582690226@163.com (T.L.); xulijingsx@hotmail.com (L.X.); cyf2341986@163.com (Y.C.); sxndcmc@163.com (M.C.); 2 Shanxi Research Center for Engineering Technology of Edible Fungi, Taigu, Jinzhong 030801, China; 3 Shanxi Key Laboratory of Edible Fungi for Loess Plateau, Taigu, Jinzhong 030801, China

**Keywords:** *Morchella sextelata*, *Pseudodiploöspora longispora*, infestation process, response mechanism, ultrastructure

## Abstract

Morels (*Morchella* spp.) are valuable and rare edible mushrooms with unique flavors and high nutritional value. White mold disease occurring during cultivation has seriously affected the quality and yield of morels in China. In this study, the fungus causing white mold disease in morels was isolated, purified, and identified as *Pseudodiploöspora longispora* by morphology and molecular biology. In addition, research has shown that *P. longispora* infection causes wrinkled and rupturing asci, loosened cell walls, and obvious membrane breakage accompanied by severe cytoplasmic leakage in *M. sextelata*. Interestingly, research has shown that infection with *P. longispora* can induce the production of an unknown substance in the cells of *M. sextelata*, which accumulates on the cell membrane, leading to membrane breakage. Furthermore, integrated transcriptomics–proteomics analysis revealed the response mechanism of *M. sextelata* to *P. longispora* infection. The results indicate that DEGs and DEPs can be significantly enriched in pathways involved in oxidoreductase activity; peroxisomes, lipid transport, and metabolism; cell wall assembly; and integral components of membranes. Further electron microscopy analysis clarified the important role of changes in the cell membrane and cell wall in the response of mycelia to biological stress. This study clarified the response mechanism of *M. sextelata* to *P. longispora*, laying a foundation for further clarifying the infection mechanism of *P. longispora*.

## 1. Introduction

Morels (*Morchella* spp.), named for their sheep belly-like shape formed by the uneven pileus [1], are famous and rare fungi for both food and medicine worldwide. Morels are rich in polysaccharides, phenolic compounds, amino acids, carotenoids, and other active ingredients. In addition to lowering blood glucose and blood lipids, the beneficial biological properties of morels include immunomodulatory, antitumor, anti-inflammatory, and antioxidant properties, and gut flora regulation [2]. Indoor cultivation has drawn much attention globally because of its tremendous economic potential. The success of indoor cultivation was first reported in 1982 by Ower [3]. After more than a century of research, field morels have been successfully cultivated for commercial purposes [4]. The scale of cultivation of morel mushrooms in China has increased annually. The *Morchella* species currently cultivated in China include *M. sextelata*, *M. importuna*, and *M. septimelata* [5]. Since 2012, the Chinese morel mushroom cultivation industry, exemplified by Sichuan Province, has been developing rapidly [6]. Unfortunately, with the development of the cultivation industry, fungal infections have become the main factor affecting the yield and quality of morels. Currently, various fungal diseases have been reported during the cultivation of morels, such as white mold disease (caused by *Pseudodiploöspora longispora* or *Paecilomyces penicillatus*) [7,8], stipe rot disease (*Fusarium incarnatum*, *Fusarium equiseti*) [9], cobweb disease (*Cladobotryum mycophilum*, *Cladobotryum protrusum*) [10,11], white rot disease (*Aspergillus* sp.) [12], stalk rot (*Fusarium nematophilum*) [13], and rot disease (*Clonostachys rosea*, *Lecanicillium aphanocladii*, *Trichoderma atroviride*) [14,15,16].

White mold disease commonly occurs in the main cultivation areas of morel mushrooms in China. It has become one of the most important diseases in the cultivation and production of morels, with the highest incidence in cultivation [17]. The disease is characterized by white, velvety hyphae on infected fruiting bodies, which expand rapidly at high temperatures (>25 °C) and high humidity (>90%), leading to wilting, rotting, deformities, and severe damage, which may result in the death of morel mushrooms. The first finding of disease caused by *P. longispora*, which mainly infects the pileus of fruiting bodies, was reported in Chongqing, China [7]. The annual incidence of white mold can reach 80% annually due to the rapid spread of large numbers of conidia around the cultivation area [17]. This is a potential risk to food safety and has been one of the major issues threatening the sustainable cultivation of morel mushrooms. Therefore, effective prevention and control methods for white mold disease are urgently needed.

Many environmental conditions are important for the prevention and control of disease infections in edible mushrooms. However, common strategies are not very effective in controlling the white mold of cultivated morels. Therefore, clarifying disease-causing mechanisms and cultivating disease-resistant varieties are effective ways to control diseases. The main mechanisms by which pathogenic fungi invade edible mushrooms include the mechanical action of mycelia, the toxic or catabolic action of secondary metabolites (toxins, cell wall degrading enzymes), and the action of immune substances that detoxify edible mushrooms. Secondary metabolites are important determinants of the pathogenicity of pathogenic fungi [18]. Peptidaibols are virulence factors that contribute to *P. longispora* invasion of *Morchella* [19]. Genomic characterization and comparative genomic analyses have been performed to reveal the evolution and related pathogenic genes of *P. longispora* through genomic characterization and comparative genomic analyses, which have provided valuable insights into the *P. longispora* genome, contributing to pathogenicity studies and drug development [20].

In recent years, the genomes of many *Morchella* species, including *M. sextelata*, *M. importuna*, and *M. septimedata*, have been published, laying the foundation for basic research on *Morchella* [21,22,23]. Current research indicates that the pathogenic mechanism of pathogenic fungi is gradually becoming clearer [24,25,26]. However, there have been no relevant reports on the response mechanism of *M. sextelata* to pathogenic fungi. This study first purified and identified the pathogen causing white mold disease. On this basis, the response pathways of *M. sextelata* to pathogenic fungi were further analyzed, providing theoretical support for the development of effective white mold control strategies.

## 2. Materials and Methods

### 2.1. Collection of Diseased White Mold M. sextelata and Isolation of the Pathogenic Fungus

White mold disease samples were collected for pathogen isolation from an *M. sextelata* cultivation greenhouse in Taigu County, Jinzhong City, Shanxi Province, China. Specifically, the surface of the fruiting bodies was rinsed with sterile water, and then the white mycelia at the diseased spots were placed on potato dextrose agar (PDA) medium and cultivated at 25 °C in the dark. Then, the pathogen was purified and cultured several times by removing the edge of the mycelia, and the purified pathogenic strains were stored at 4 °C. The remaining samples were stored at −80 °C.

### 2.2. Pathogenicity Test and Microscopic Observation

According to previous research methods, the isolated and purified pathogenic fungi were inoculated on PDA at 25 °C and cultured for 14 d until spore production, after which a spore suspension (1.0 × 10^6^ spores mL^−1^) was prepared [27]. The surface of the *M. sextelata* was sterilized with 75% ethyl alcohol, a wound of approximately 5 mm was cut on the cap of the *M. sextelata* with a sterile scalpel, and 200 μL of spore suspension was added to the wound. Equal amounts of sterile water were added to the scratches as a blank control. The *M. sextelata* treated were covered with plastic bags for humidity preservation. The onset of disease was observed and recorded every day. After 5 days, the pathogen was re-isolated from infected *M. sextelata* and characterized by morphological and phylogenetic analyses to test Koch’s postulates [7].

The samples (1–2 mm^3^) were completely submerged in 2.5% glutaraldehyde solution and stored at 4 °C overnight. The samples were rinsed with 0.1 M, pH 7.0 phosphate buffer solution (PBS) after the 2.5% glutaraldehyde fixative was removed. The samples were then dehydrated, critical point-dried, and coated, as previously reported [28]. The treated samples were observed via scanning electron microscopy (SEM) (Regulus 8100, Hitachi, Tokyo, Japan). The samples (1–2 mm^3^) were placed into 2.5% glutaraldehyde and fixed at 4 °C for storage. The glutaraldehyde solution was removed, and the samples were rinsed three times with PBS for 15 min each time. The samples were fixed with 1% osmium for 1–2 h, and then rinsed with PBS three times. The samples were then dehydrated with ethanol solution and finally treated with pure acetone for 20 min. The samples were treated with a mixture of embedding agents and acetone. Pure embedding agent was used to treat the samples overnight, and the osmotically treated samples were embedded and heated at 70 °C overnight to obtain the embedded samples [29]. The samples were sectioned in an ultrathin microtome (EMUC7, Leica, Wetzlar, Germany) to obtain 70–90 nm sections, which were stained with lead citrate solution and uranyl acetate 50% ethanol saturated solution for 5–10 min, and then air-dried for visualization via transmission electron microscopy (TEM) (JEM1400, Hitachi, Tokyo, Japan) for observation.

### 2.3. Identification of Pathogens Causing White Mold Disease in M. sextelata

For morphological identification, the pathogen isolates were cultured on PDA at 25 °C for 10 days. Microstructures, including mycelia, conidia, conidiophores, and chlamydospores, including conidiophores and conidial size, were studied using optical microscopy (Eclipse Ni-U, with a Ri2 camera, Nikon, Tokyo, Japan) [30]. The genomic DNA of pathogen isolates was extracted using the E.Z.N.A.^®^ Forensic DNA Extraction Kit (Omega Bio-Tek, Norcross, GA, USA). The primer pair ITS4 (5′-TCCTCCGCTTATTGATATGC-3′)/ITS5 (5′-GGAAGTAAAAGTCGTAACAAGG-3′) was used for amplifying the internal transcribed sequence (ITS) region [31]. The PCR mixture contained 13 μL of 2 × Easy Taq PCR SuperMix (+dye), 1 µL of each primer, 2 µL of genomic DNA, and ddH_2_O for a total reaction volume of 25 µL. PCR analysis was conducted in a Bio-Rad T100 Thermal Cycler (Bio-Rad Laboratories Inc., Hercules, CA, USA). The amplification conditions were as follows: 1 cycle at 95 °C for 3 min; 34 cycles at 94 °C for 30 s, 53 °C for 30 s, and 72 °C for 30 s; and 1 cycle at 72 °C for 10 min. Amplification products were detected using gel electrophoresis on 1% agarose gels stained with Gel Green. The successfully amplified products were cleaned using an E.Z.N.A.^®^ Cycle-Pure Kit (Omega Bio-Tek) and sequenced at Tsingke Biotechnology Co., Ltd. (Xi’an, China). The sequencing results were assembled with DNAMAN 9.0 software (https://www.lynnon.com) (accessed on 12 October 2023), and the assembled results were subsequently aligned by BLAST (Basic Local Alignment Search Tool) on the NCBI website (https://blast.ncbi.nlm.nih.gov/Blast.cgi) (accessed on 25 October 2023) to construct a phylogenetic tree using MEGA X [32,33].

### 2.4. Inhibitory Effect of the Pathogenic Fungus on M. sextelata on Plates

The inhibitory effect of water-soluble substances from pathogenic fungi on the growth of the mycelia of *M. sextelata* was detected using the plate standoff method [34] and fermentation liquid inhibition tests. *M. sextelata* cakes and pathogenic fungal cakes (diameter = 6 mm) were placed on each side of the PDA plate. Two pathogenic fungal cakes (diameter = 6 mm) were placed on each side of plates covered with *M. sextelata* mycelia. All of the plates were incubated at 25 °C in the dark under static and constant humidity conditions for 5 days of cultivation, and the mycelial growth of the two fungal strains in each treatment was observed. Pathogenic fungal mycelia 6 mm in diameter were inserted into a 250 mL conical flask containing 100 mL of potato dextrose broth (PDB) and incubated at 25 °C and 150 rpm/min for 6 days of oscillatory cultivation. The mycelia were filtered to obtain fermentation broth, which was subsequently filtered through a sterile 0.22 μm filter to obtain a sterile fermentation broth. After the PDA solid medium was autoclaved, it was cooled to approximately 55 °C, and fermentation broth was added, 5 mL of filtrate per 100 mL of PDA medium, mixed well, and poured into the plates to produce PDA plates containing 5% filtrate. In the control group, distilled water was used instead of fermentation broth to make the culture medium. The activated 6 mm diameter mycelia cakes of *M. sextelata* were placed on PDA supplemented with fermentation broth and incubated at 18 ℃ in the dark to observe the growth of *M. sextelata*. Based on the observed phenotypic changes, mycelial samples from the fermentation broth-treated (MT) and control groups (CK) were scraped and analyzed via transcriptomics and proteomics.

### 2.5. Effect of Nutrients from M. sextelata on Pathogenic Fungi

To explore whether the nutrients of *M. sextelata* can promote the growth of pathogenic fungi, dried *M. sextelata* were crushed with a pulverizer and sieved through a 100-mesh sieve. The powder of *M. sextelata* was added to PDA to final concentrations of 1% and 2%, with PDA medium used as a control group. Activated pathogen cakes (6 mm diameter) were placed on the three kinds of plates. After 10 days of incubation at 25 °C in the dark, the growth of the pathogen was observed, and the colony diameter was measured.

### 2.6. Transcriptome Sequencing

Mycelial samples of *M. sextelata* scraped from the MT group and CK group were sent to Majorbio Biopharm Technology Co., Ltd. (Shanghai, China) and sequenced on the Nova Seq6000 platform (Illumina, San Diego, CA, USA). Total RNA was extracted from the tissue using TRIzol^®^ Reagent (Majorbio Biomedical Technology Co., Ltd., Shanghai, China) according the manufacturer’s instructions. The differentially expressed genes (DEGs) were analyzed based on a |log2FC| ≥ 1 and *p*-adjusted < 0.05. Gene ontology (GO) enrichment and Kyoto Encyclopedia of Genes and Genomes (KEGG) enrichment analyses were performed by Goatools and R scripts, respectively, and the Majorbio Cloud Platform was utilized to analyze the data (Majorbio Biomedical Technology Co., Ltd., Shanghai, China, www.majorbio.com) (accessed on 20 January 2024).

### 2.7. Proteomic Sequencing

The same samples used for transcriptome sequencing were used. Proteomic analysis was performed at Majorbio Biopharm Biotechnology Co., Ltd. (Shanghai, China), on an Orbitrap Astral high-resolution mass spectrometer. The differentially expressed proteins (DEPs) were analyzed based on a |log2FC| >= 1 and *p*-adjusted < 0.05.

### 2.8. Quantitative PCR (qPCR) Analysis

The qPCR analysis was performed to confirm the validity of the RNA-seq results. The qPCR amplification program was as follows: amplification at 95 °C for 3 min, amplification at 95 °C for 3 s, amplification at 60 °C for 32 s, 40 cycles, and amplification at 72 °C for 3 s. Supplementary Table S1 lists the primers used for qPCR. The relative expression levels of all genes were ascertained according to the 2^−∆∆CT^ method.

### 2.9. Statistical Analysis and Data Availability

Statistical analysis was performed using SPSS 25 software. The independent samples *t*-test was used to examine the data, which are presented as the means ± SEs. A *p*-value less than 0.05 was considered to indicate statistical significance. For figure analysis, GraphPad Prism 8 and Photoshop 2023 were utilized. The reference genome of *M. sextelata* can be downloaded from https://www.ncbi.nlm.nih.gov/genome/86229?genome_assembly_id=748597 (accessed on 18 January 2024).

## 3. Results

### 3.1. Pathogen Isolation and Identification

**Figure 1:**
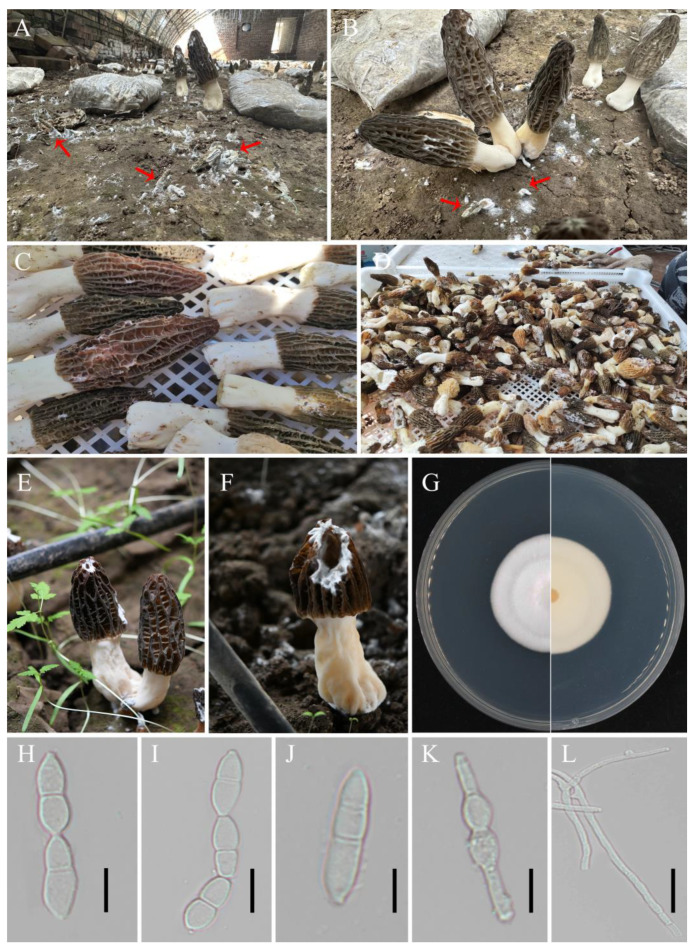
Occurrences of fungal disease in *M. sextelata* ascocarps cultivated outdoors and the morphological characteristics of *M. sextelata* fungal disease. (**A**–**D**) Disease-infected young fruiting bodies (red arrow) of *M. sextelata*. (**E**,**F**) Wilting and shriveling of naturally infested *M. sextelata*. (**G**) Colony of the pathogenic isolate grown on PDA at 25 °C for 14 days and on the reverse side. (**H**–**J**) Conidia of the pathogenic isolate. (**K**) Chlamydospores of the pathogenic isolate. (**L**) Hyphae of the pathogenic isolate. Scale bars: (**H**–**L**) = 10 μm.

White mold disease spreads rapidly and lacks an effective means of control, which can result in a loss of quality and quantity and lead to economic losses in commercial morel cultivation (Figure 1A–D). White mold disease occurs mainly on the cap of fruiting bodies. In this study, rotting of the pileus of *M. sextelata* was observed (Figure 1E,F). After 8 days of cultivation on PDA plates, the colony diameter of the pathogenic fungi was 3–4 cm. The colonies were projected and white, with smooth edges and sparse to medium aerial mycelia (Figure 1G). The conidia were colorless and transparent, ellipsoidal or citriform, 4.5–5.5 × 15–18 μm, and had 1–3 septates (predominantly 1 septate) (Figure 1H–J). Chlamydospores in terminal or intercalary clusters of 2–10 cells are produced from vegetative hyphal cells (Figure 1K). With a diameter ranging from 6.3 to 11.5 μm and a smooth or verrucose surface, they were spherical, usually released in clusters, short or long chains, and occasionally in pairs or individually. The hyphae had a diameter of 2–4 μm and were smooth, septate, branched, and hyaline (Figure 1L).

**Figure 2:**
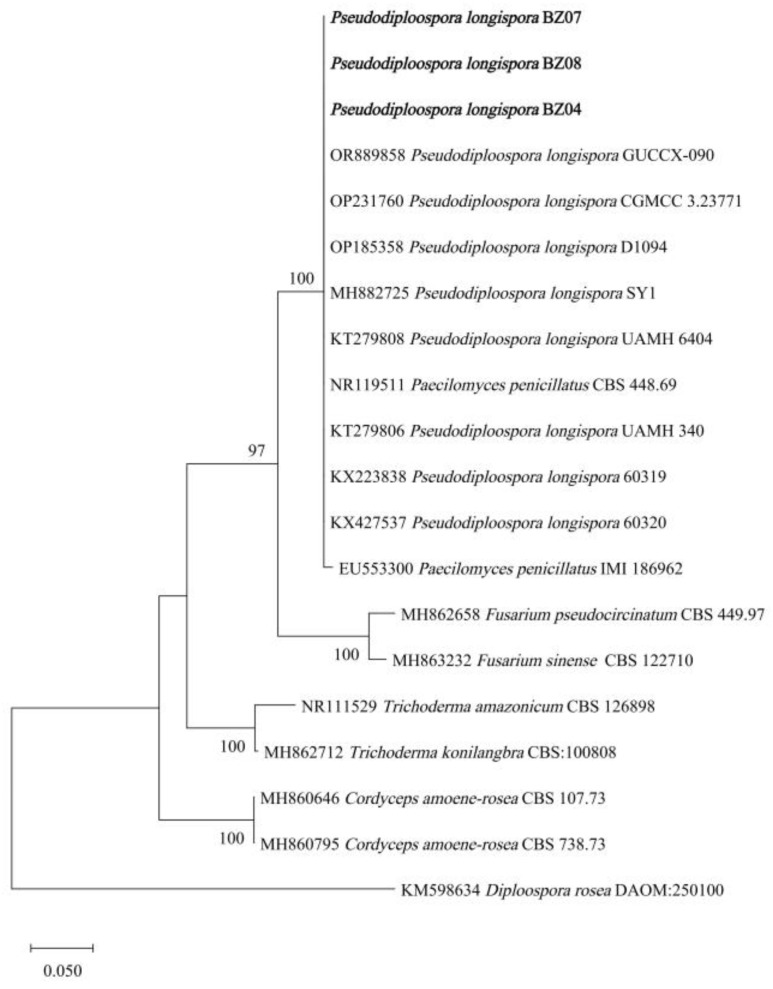
ML phylogenetic tree based on the ITS sequences. The bolded parts are the sequences of the isolates in this study.

The internal transcribed spacer (ITS) region of ribosomal DNA (rDNA-ITS) was sequenced and analyzed. The ITS fragments of strains BZ04, BZ07, and BZ08 were 511 bp, 516 bp, and 491 bp (GenBank accession numbers PP935354, PP935355, and PP935354), respectively. After searching and comparing the sequences with the NCBI database using BLAST, it was discovered that they had more than 99.80% similarity with those of *Pseudodiploospora longispora* and *Paecilomyces penicillatus*. The phylogenetic tree showed that the sequences of strains BZ04, BZ07, and BZ08 were in the same branch as those of *P. longispora* and *P. penicillatus* (Figure 2). The conidia of *P. longispora* have septates, whereas those of *Paecilomyces* spp. have no septates [35]. The conidia of *P. penicillatus* have no septates, whereas the pathogenic isolates of this study had 1–3 septates [7]. Therefore, based on the morphological traits and rDNA-ITS sequencing analysis results, *P. longispora* was finally determined to be the fungus causing pileus rot on cultivated *M. sextelata*.

### 3.2. Pathogenicity Testing and Effect of P. longispora Infestation on the Micromorphology of M. sextelata

**Figure 3:**
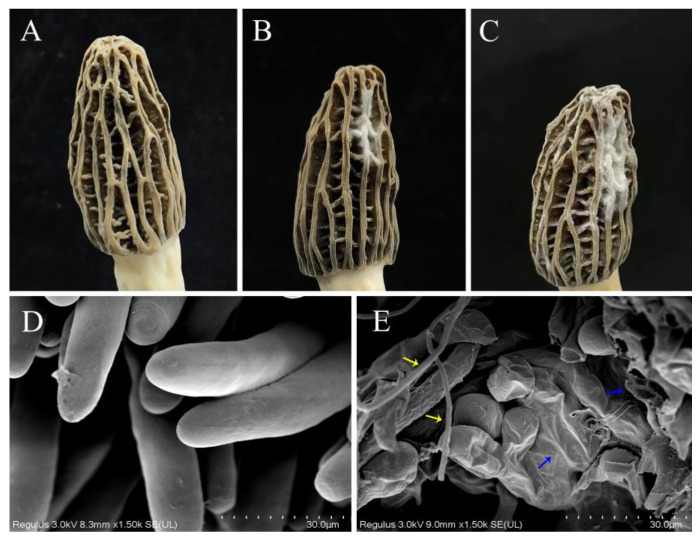
Pathogenicity testing and SEM observations of the fruiting body of *M. sextelata*. (**A**) Mycelial tissue treated with sterile water showed no symptoms. (**B**) Symptoms observed 3 days after wound inoculation of healthy *M. sextelata* with fungal suspensions. (**C**) Symptoms observed 6 days after wound inoculation of healthy *M. sextelata* with fungal suspensions. (**D**) Morphology of the asci of healthy *M. sextelata* via SEM. (**E**) Morphology of the asci of the disease-infected *M. sextelata*; hyphae of *P. longispora* (yellow arrow) crossing the fruiting body of *M. sextelata*, and the asci were crumpled and cracked (blue arrow).

In the early stage of the disease, irregular lesions and white fluffy mycelia appeared on the cap. With the continued infection of the pathogen, the mold layer continued to expand, leading to shrinkage and drying of the infected area. The cap undergoes decay and even perforation, which severely affects the growth and development of fruiting bodies and ultimately leads to wilting and death. A spore suspension of the isolated strain was made for pathogenicity testing. Pathogenicity results showed that 3 days after inoculation with the spore suspension, typical dense, white, thick mycelia were visible at the inoculation site (Figure 3B). After 6 days, the lesions expanded to form a large layer of white mold (Figure 3C), while the lesions in the control group did not significantly change (Figure 3A). Symptoms from artificial inoculation resembled those seen in the wild. To satisfy Koch’s postulates, re-isolation from infected fruiting bodies was carried out, and the pathogen was confirmed to be in alignment with the inoculated isolate based on morphological characteristics.

To determine the effect of pathogenic fungal infection on the morphology and ultrastructure of *M. sextelata*, SEM was used. Healthy *M. sextelata* asci were cylindrical, full, and neatly arranged (Figure 3D). However, the mycelia of *P. longispora* invaded the *M. sextelata* along the interstitial space, causing the wall of the asci to shrink and ultimately leading to its rupture (Figure 3E).

### 3.3. The Response of M. sextelata to P. longispora Infection

**Figure 4:**
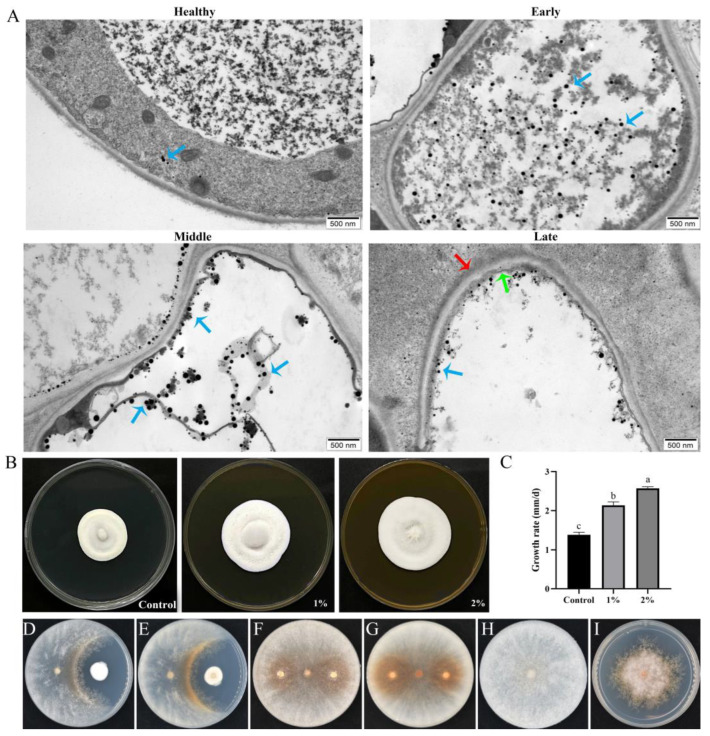
The process by which *P. longispora* infests the cells of *M. sextelata* and *M. sextelata* promotes the growth of *P. longispora*. (**A**) Process by which *P. longispora* infests *M. sextelata* cells. Complex of osmium tetroxide with a substance in the cell (blue arrow), cell wall (red arrow), and cell membrane (green arrow). (**B**) Colony morphology of *P. longispora* on media supplemented with different concentrations of *M. sextelata* powder for 10 days. (**C**) Growth rates of *P. longispora* on media supplemented with different concentrations of *M. sextelata* powder. Different letters indicate significant differences for the comparison of samples (*p* < 0.05 according to Duncan’s test). (**D**,**E**) Confrontation cultures of *P. longispora* and *M. sextelata* on PDA media. (**F**,**G**) *P. longispora* cultured on mycelium of *M. sextelata*. (**H**) *M. sextelata* cultured on PDA for 9 days. (**I**) *M. sextelata* cultured on fermentation broth for 9 days.

Compared with those of healthy cells, the ultrastructures of the cells of *M. sextelata* with different degrees of infestation changed significantly (Figure 4A). The healthy cells had an orderly distribution of internal organelles and a small number of punctate substances. In the early infection stage, the number of punctate substances increased, and the substances were evenly dispersed in the *M. sextelata* cells. In the middle stage, the cell contents gradually flowed out, and the punctate substances were concentrated on the membrane. In the late stage, the cell wall became loose, the cell membrane was severely disrupted, and the number of punctate substances attached to the membrane obviously decreased. This dot-like substance exists in small quantities and is distributed irregularly in the healthy state but increases significantly after infestation by the pathogenic fungus and gradually concentrates on the membrane inside.

This study showed that *P. longispora* infestation leads to the rupture of *M. sextelata* cell membranes and the outflow of contents. To investigate whether *M. sextelata* can promote the growth of *P. longispora*, a dry powder of *M. sextelata* was added to the culture medium of *P. longispora*. The results demonstrated that the mycelial growth rate reached a maximum on medium supplemented with 2% *M. sextelata* powder and reached a minimum on the control PDA medium (Figure 4B,C). This indicates that *P. longispora* can utilize the nutrients of *M. sextelata* to promote its own growth.

A clear inhibition cycle between *P. longispora* and *M. sextelata* was observed (Figure 4D). The isolated strains significantly inhibited the mycelial growth of *M. sextelata* without touching *M. sextelata*. *P. longispora* can significantly inhibit the growth of *M. sextelata* by producing soluble substances. A significant brownish color at the antagonist line was observed on the back of the plate (Figure 4E). The *P. longispora* were inoculated onto plates covered with *M. sextelata* mycelia, and a distinct brown color also appeared around the inoculation site (Figure 4F,G). At the junction of the two strains, obvious brown pigment deposition can be observed. In the inhibition test of the *P. longispora* fermentation solution, the *M. sextelata* cultivated on the fermentation solution media were obviously inhibited compared with those cultivated on the control PDA medium (Figure 4H,I). Moreover, the mycelia on the medium containing fermentation broth were thinner and finer.

### 3.4. Functional Enrichment Analysis of DEGs

**Figure 5:**
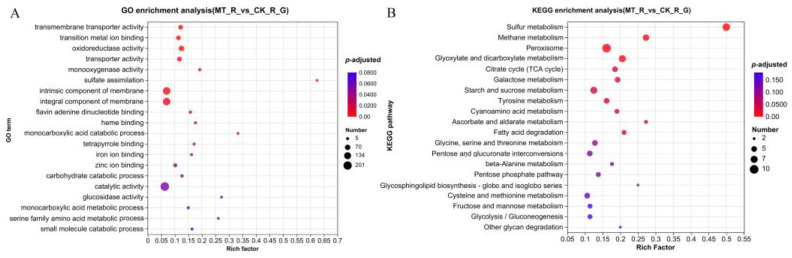
Functional enrichment analyses of DEGs in *M. sextelata*. (**A**) GO enrichment analyses of DEGs. GO term enrichment *p*-values are indicated on the *x*-axis. BP, biological process; MF, molecular function; CC, cellular component. (**B**) KEGG enrichment analyses of DEGs. The x-axis represents the enrichment factor. The size of the dot indicates the number of DEGs involved in the pathway. The color bars on the right represent the *p*-values of the KEGG pathway.

With a |log2FC| ≥ 2 and *p*-adjusted < 0.05, a total of 491 DEGs were identified in diseased *M. sextelata*, including 134 upregulated genes and 357 downregulated genes, compared to healthy controls. To clarify the functional enrichment of DEGs in *M. sextelata*, GO category analysis was applied. A total of 122 GO terms were enriched in the DEGs. Among them, transmembrane transporter activity (GO:0022857), transition metal ion binding (GO:0046914), oxidoreductase activity (GO:0016491), transporter activity (GO:0005215), monooxygenase activity (GO:0004497), and iron ion binding (GO:0005506) were in the molecular function category; sulfate assimilation (GO:0000103), monocarboxylic acid catabolic process (GO:0072329), and carbohydrate catabolic process (GO:0016052) were in the biological processes category; and intrinsic component of membrane (GO:0031224) and integral component of membrane (GO:0016021) were in the cellular component category among the top 20 GO terms (Figure 5A). DEGs related to oxidoreductase activity were screened, including mitochondrial peroxiredoxin, manganese superoxide dismutase, and catalase B. The highest numbers of DEGs enriched in the intrinsic component of membrane (GO:0031224) and integral component of membrane (GO:0016021) terms were 151 and 150, respectively. Several DEGs involved in integral components of the membrane, such as iron transport multicopper oxidase and peroxisomal membrane protein 2, were screened for analysis.

KEGG pathway enrichment analysis was conducted in detail. The DEGs were mapped to 64 KEGG pathways, including 11 significantly enriched pathways: sulfur metabolism (ko00920), methane metabolism (ko00680), peroxisome (ko04146), glyoxylate and dicarboxylate metabolism (ko00630), citrate cycle (TCA cycle) (ko00020), galactose metabolism (ko00052), starch and sucrose metabolism (ko00500), tyrosine metabolism (ko00350), cyanoamino acid metabolism (ko00460), ascorbate and aldarate metabolism (ko00053), and fatty acid degradation (ko00071) (Figure 5B). Furthermore, peroxisomes (ko04146) were enriched with the greatest number of DEGs. Catalase B was significantly downregulated, and manganese superoxide dismutase was significantly upregulated by pathogen infection.

### 3.5. Functional Annotation and Enrichment Analysis of DEPs

**Figure 6:**
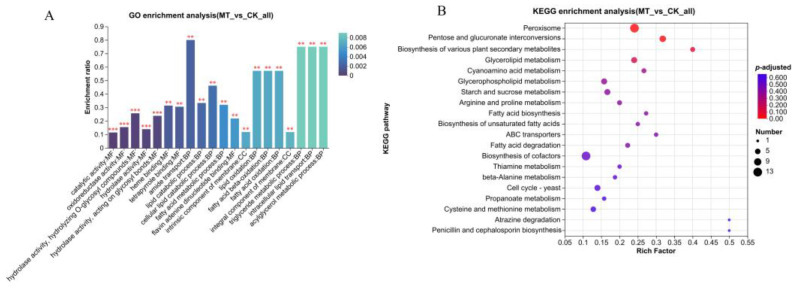
Functional enrichment analyses of DEPs in *M. sextelata*. (**A**) GO enrichment analyses of DEPs. Column color gradients indicate the significance of enrichment; *p* or FDR < 0.001 is labeled ***, and *p* or FDR < 0.01 is labeled **. (**B**) KEGG enrichment analyses of DEPs.

For the identification of differentially expressed proteins (DEPs), FC > 2 or <0.5 and *p*-value < 0.05 were used as criteria. In total, we identified 447 DEPs, including 150 significantly upregulated proteins and 297 significantly downregulated proteins. A total of 88 GO terms were enriched in the DEGs. GO enrichment analyses revealed significant enrichment of the top 20 GO terms (Figure 6A), including catalytic activity (GO:0003824), oxidoreductase activity (GO:0016491), three hydrolase activities (GO:0004553, GO:0016787, GO:0016798), heme binding (GO:0020037), tetrapyrrole binding (GO:0046906), and flavin adenine dinucleotide binding (GO:0050660) in the category of molecular function; amide transport (GO:0042886), lipid catabolic process (GO:0016042), cellular lipid catabolic process (GO:0044242), fatty acid metabolic process (GO:0006631), lipid oxidation (GO:0034440), fatty acid beta-oxidation (GO:0006635), fatty acid oxidation (GO:0019395), triglyceride metabolic process (GO:0006641), and acylglycerol metabolic process (GO:0006639) in the biological processes category; and intracellular lipid transport (GO:0032365), intrinsic component of membrane (GO:0031224), and integral component of membrane (GO:0016021) in the cellular component category. KEGG pathway analysis revealed two important enrichment pathways: peroxisome (ko04146) and pentose and glucuronate interconversions (ko00040) (Figure 6B). Consistent with the RNA-seq results, the peroxisome (ko04146) pathway was the most enriched pathway among the DEPs. Heatmap analysis revealed that all 13 DEPs enriched in the peroxisome pathway were downregulated (Supplementary Figure S3).

### 3.6. Combined Transcriptomics and Proteomics Analysis

**Figure 7:**
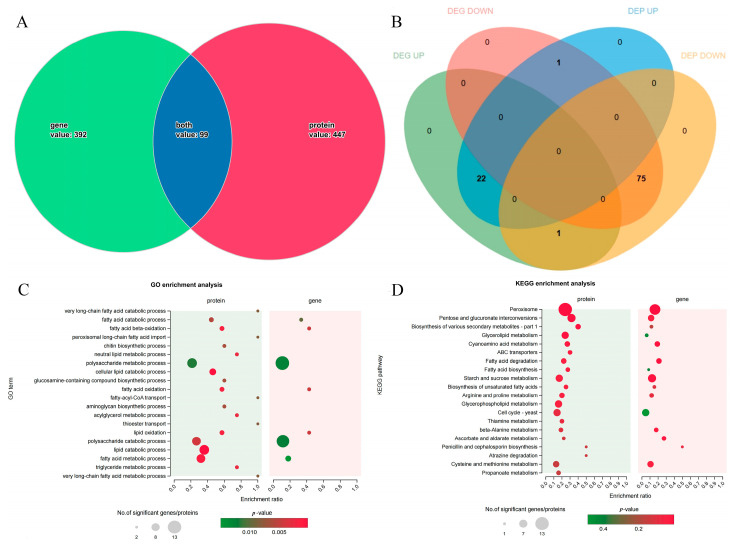
Combined transcriptomic and proteomic analysis. (**A**) Joint Wayne analysis of total DEGs/DEPs. (**B**) Joint Wayne analysis of shared factors. (**C**) GO enrichment analyses. (**D**) KEGG enrichment analyses.

Venn analysis revealed that the proteins encoded by the 99 DEGs were DEPs (Figure 7A). Among them, 97 DEGs, including 22 upregulated and 75 downregulated genes, showed the same change patterns as their encoded proteins (Figure 7B, Supplementary Tables S2 and S3). The changes in the expression of these two genes were opposite to those in the expression of their encoded proteins. GO functional enrichment analysis of 99 DEGs revealed that fatty acid catabolic process (GO:0006631), fatty acid beta-oxidation (GO:0006635), polysaccharide metabolic process (GO:0005976), fatty acid oxidation (GO:0019395), lipid oxidation (GO:0034440), polysaccharide catabolic process (GO:0000272), and fatty acid metabolic process (GO:0006631) were significantly enriched at both the mRNA and protein levels (Figure 7C). The expressions of two genes’ (carboxylesterase and glycerol-1-phosphate phosphohydrolase 2) encoded proteins were jointly upregulated, and the expressions of five genes (peroxisomal hydratase-dehydrogenase-epimerase, mevalonyl-coenzyme A hydratase, sterol carrier protein 2, acyl-CoA ligase, and probable acyl-CoA dehydrogenase), whose encoded proteins are involved in lipid transport and metabolism, were jointly downregulated. The 22 upregulated DEGs coregulated with encoded proteins were significantly enriched in 36 GO terms, with 7 terms associated with the cell wall (Supplementary Figure S1). GO enrichment analyses were performed on 75 jointly downregulated genes (Supplementary Figure S2). Among them, 1,3-β-glucanosyltransferase was jointly upregulated in cell wall assembly, and endopolygalacturonase B was likely to jointly downregulate cell wall organization.

According to the results of the combined KEGG pathway enrichment (Figure 7D), DEGs and DEPs were mainly enriched in peroxisomes (ko04146), pentose and glucuronate interconversions (ko00040), biosynthesis of various secondary metabolites (ko00999), and fatty acid degradation (ko00071). KEGG pathway enrichment analysis of the 22 DEGs whose expression was upregulated revealed that genes involved in beta-alanine metabolism (ko00410) and arginine and proline metabolism (ko00330) were significantly enriched. The 75 downregulated genes that exhibited the same change patterns as their encoded proteins were significantly enriched in pentose and glucuronate interconversions (ko00040), peroxisomes (ko04146), beta-alanine metabolism (ko00410), fatty acid degradation (ko00071), and penicillin and cephalosporin biosynthesis (ko00311).

### 3.7. Validation of Candidate Genes by qPCR Analysis

**Figure 8:**
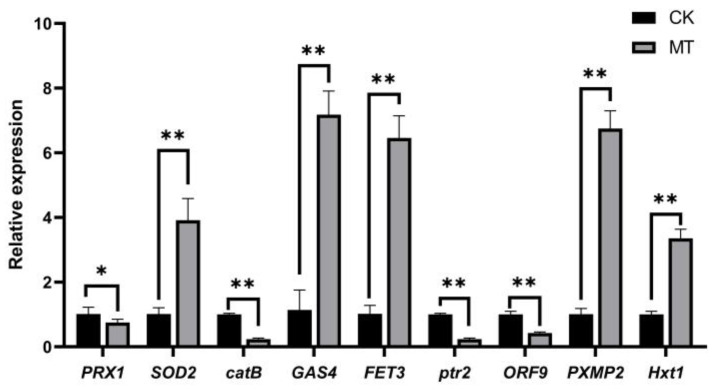
Validation of candidate genes via qPCR. CK: control group; MT: fermentation broth-treated group. Asterisk denotes statistically significant differences, * *p* < 0.05; ** *p* < 0.01.

To verify the reliability of the RNA-seq results, 9 key DEGs were selected from three important terms and pathways, oxidoreductase activity, peroxisomes, cell wall and integral components of membrane, and subjected to qPCR analysis (Table 1). The results demonstrated that the relative expressions of these genes matched the RNA-seq data (Figure 8). This result confirmed the validity of the RNA-seq data.

In summary, integrated transcriptomics–proteomics analysis revealed that metabolic pathways related to the cell wall and cell membrane of *M. sextelata* play important roles in the response to *P. longispora*.

### 3.8. Changes in the Cell Membrane and Wall of M. sextelata Are Markers in Response to P. longispora Infection

**Figure 9:**
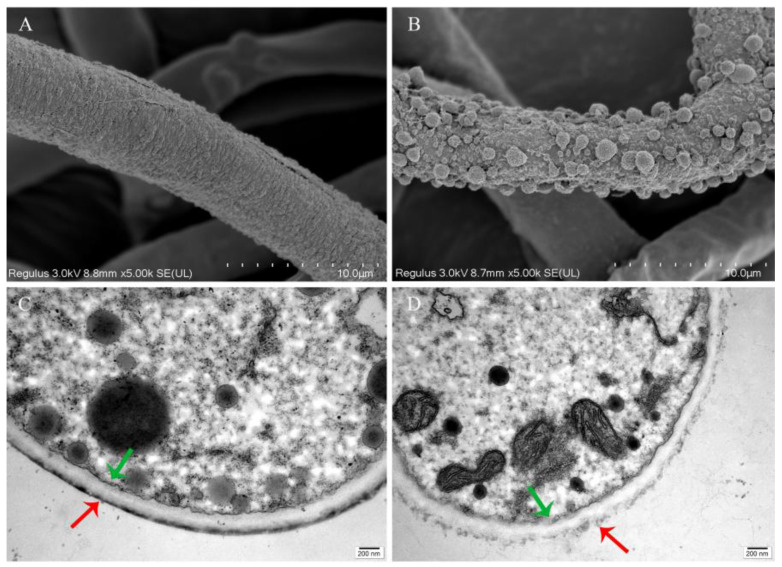
Effects of *P. longispora* metabolites on *M. sextelata* mycelial morphology. (**A**) *M. sextelata* mycelia surface of CK group. (**B**) *M. sextelata* mycelia surface of MT group. (**C**) *M. sextelata* mycelial cells of CK group. (**D**) *M. sextelata* mycelial cells of MT group. Cell wall (red arrow) and membrane (green arrow). CK: control group; MT: fermentation broth-treated group.

Furthermore, electron microscopy was used to observe the effects of *P. longispora* on the cell membrane and cell wall of *M. sextelata* mycelia. The results showed that there were a large number of spherical attachments on the surface of the *M. sextelata* mycelia in the MT group (Figure 9B), whereas none were found on the surface of the *M. sextelata* mycelia in the control group (Figure 9A). Combined with the morphology of mycelia on plates, it was speculated that the spherical attachments might be caused by the rapid senescence of the mycelia. TEM observations of mycelial cells showed that the *P. longispora* fermentation filtrate resulted in loosening of the mycelial cell wall and rupture of the cell membrane (Figure 9D).

## 4. Discussion

White mold is one of the most prevalent diseases of morel mushrooms and is widespread throughout China. The production of morels in China is seriously threatened by large-scale and extremely invasive pathogenic fungi; however, little research has been conducted on the pathogenic mechanisms of this fungus. In this study, white mold samples were collected from the cultivation area of *M. sextelata* in Taigu County, Shanxi Province. The pathogen was identified as *P. longispora* by morphology, molecular biology, and pathogenicity testing. White mold disease in cultivated *M. importuna* caused by *P. longispora* (previously known as *Diploospora longispora*) was first reported in Chongqing, China [7]. In this study, when performing the dural culture method, a significant brownish color at the antagonist line was observed on the back of the plate. A similar pigment deposition phenomenon was also reported in *Lentinus edodes*, which is thought to be a defense measure [43]. Therefore, brown pigmentation may also be a defense measure of *M. sextelata* against *P. longispora*.

The cell wall is an important physical defense barrier for plants and fungi and plays an important role in resisting infection by pathogenic fungi [44]. The fungal cell wall mainly consists of an outer layer of glycoproteins and an inner skeletal layer of glucans and chitin [45]. In plants, maintaining cell wall integrity (CWI) is crucial for initiating and tracking defense responses [46]. Upon invasion by pathogens, the CWI system perceives changes in cell wall status to activate defense responses [47]. The modification of the chemical composition or structure of the cell wall is an active defense response against pathogen invasion [48]. In this study, cytological analysis revealed that after infection with *P. longispora*, the mycelial cell wall of *M. sextelata* was loose. In addition, chitin synthase I and 1,3-β-glucanosyltransferase activity were upregulated in *M. sextelata*, suggesting that these genes may be involved in cell wall remodeling [13]. Previous studies have also shown that pathogen infection can damage the cell walls of plants or fungi. Infection by *Magnaporthe oryzae* can cause damage to the cell wall of rice leaves, triggering a series of defense responses [49]. *Lycium barbarum* responds to *Fusarium solani* infection by regulating the composition of the cell wall, the stability of the cytoskeleton, and the function of the cell membrane [50]. The infection of *Pseudomonas* sp. can lead to the breakdown of the *Pleurotus eryngii* cell wall, resulting in the leakage of soluble sugars from the contents of *P. eryngii* [51]. Therefore, the CWI plays a crucial role in the stress response. However, it is still unknown how the integrity of edible mushroom cell walls perceives stress and activates defense responses.

The cell membrane plays an important role in material transport, energy conversion, and information transfer between the cell and the external environment [52]. In this study, pathogen infection induced the production of an unknown substance in *M. sextelata* cells, which accumulated on the cell membrane, leading to membrane breakage accompanied by severe cytoplasmic leakage. Previous studies have shown that *Alternaria alternate* toxins can cause changes in the membrane permeability of *Citrus reticulata* cells, leading to electrolyte permeation and affecting their normal metabolism by causing changes in membrane potential [53], which is similar to the results of this study. Lipids are the main components of cell membranes and play an indispensable role in maintaining the structural integrity of the cell; excessive oxidation of lipids changes the physical properties of cell membranes and leads to destruction of the cell membrane structure [54]. DEGs and DEPs were significantly enriched in the lipid oxidation process (Figure 6). Among them, *FET3*, encoding a multicopper oxidase with ferroxidase activity, is upregulated in infected *M. sextelata*. Early research revealed that peptaibols containing Aib residues can form helical structures that allow host cell lipid bilayer membrane iron channels to extend, causing cell death and internal leakage [55]. It is speculated that the high expression of *FET3* in *M. sextelata* may be involved in the process of cell membrane rupture. In addition, transmembrane transporter activity (GO:0022857) was the most enriched GO term in the infected *M. sextelata*. In this regard, the expressions of two oligopeptide transporter genes significantly decreased during the response of *M. sextelata* to disease. Research has shown that the proteins of the oligopeptide transport family can transport their reaction substrates from the external environment or organelles to the cytosol [56]. Therefore, it can be inferred that a decrease in the expression level of oligopeptide transporter genes is a marker of cell membrane damage.

Moreover, one of the first reactions observed when cells are damaged is a shift in the reduction oxidation condition [57]. In this study, DEGs and DEPs were significantly enriched in oxidoreductase activity (GO:0016491) and peroxisomes (ko04146). *SOD2* was significantly upregulated in *M. sextelata* after stimulation by *P. longispora*. Unexpectedly, *CAT* was significantly downregulated. Previous studies have shown that *SOD* and *CAT* are significantly upregulated in *Agaricus bisporus* infected with *Lecanicillium fungicola* [37]. It can be inferred that the antioxidant system also plays an important role in the response of *M. sextelata* to *P. longispora* infection.

## 5. Conclusions

**Figure 10:**
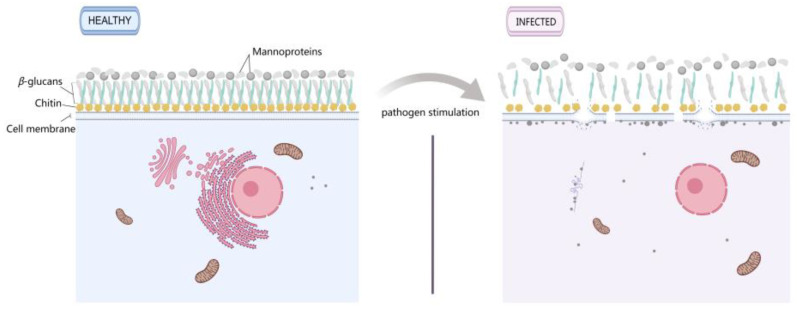
Comparison of healthy and infested *M. sextelata* cells.

In summary, white mold samples were collected from a cultivation greenhouse of *M. sextelata* in Taigu County, Shanxi Province, China. The pathogen was identified as *P. longispora* by morphology, molecular biology, and pathogenicity testing. This study clarified the response of *M. sextelata* cells to *P. longispora*. Analyses at the cytological level demonstrated that infection with *P. longispora* can induce the production of an unknown substance in the mycelia of *M. sextelata*, which accumulates on the cell membrane, leading to membrane breakage (Figure 10). Furthermore, integrated transcriptomics–proteomics analysis revealed the response mechanism of *M. sextelata* to *P. longispora* infection. A total of 491 DEGs and 447 DEPs were identified in diseased *M. sextelata*. Most of the DEGs and DEPs were related to oxidoreductase activity, peroxisomes, the cell wall, and integral components of membrane and lipid transport and metabolism processes, such as *PRX1*, *SOD2*, *catB*, *GAS4*, *FET3*, *ptr2*, *ORF9*, *PXMP2*, and *Hxt1*, which play important roles in the response to disease in *M. sextelata*. This study elucidated the mechanism underlying the response of *M. sextelata* to *P. longispora* and provided a foundation for elucidating the infection mechanism of *P. longispora*. In addition, the DEGs and DEPs screened in this study provide targets for the study of the disease resistance mechanism of *M. sextelata*.

## Figures and Tables

**Table 1:** Information on key genes for qPCR analysis.

Gene ID	Description	Gene Name	Function	Organism
gene-H6S33_012182	Mitochondrial peroxiredoxin	*PRX1*	reduces hydrogen peroxide and alkyl hydroperoxides	*Aspergillus fumigatus* [36]
gene-H6S33_004573	Manganese superoxide dismutase	*SOD2*	neutralizes superoxide anion radicals and protects cells from oxidative stress	*Agaricus bisporus* [37]
gene-H6S33_004442	Catalase B	*catB*	serves to protect cells from the toxic effects of hydrogen peroxide	
gene-H6S33_008738	1,3-beta-glucanosyltransferase	*GAS4*	involved with Gas2p in spore wall assembly	*Saccharomyces cerevisiae* [38]
gene-H6S33_005983	Iron transport multicopper oxidase	*FET3*	multicopper oxidase that oxidizes ferrous (Fe^2+^) to ferric iron (Fe^3+^) for subsequent cellular uptake by transmembrane permease Ftr1p	*Fusarium graminearum* [39]
gene-H6S33_000069	Probable peptide transporter	*ptr2*	transport nitrogen-containing substrates	*Fusarium graminearum* [40]
gene-H6S33_000010	Cytochrome P450 monooxygenase	*ORF9*	involved in the metabolism of various endogenous substrates, including fatty acids, steroid hormones, and vitamins	*Penicillium roqueforti* [41]
gene-H6S33_005547	Peroxisomal membrane protein 2	*PXMP2*	involved in pore-forming activity and may contribute to the unspecific permeability of the peroxisomal membrane	NO
gene-H6S33_006216	Hexose transporter	*Hxt1*	monosaccharide transporter and sensor	*Ustilago maydis* [42]

## Data Availability

All data generated or analyzed during this study are included in this published article and the supplementary information files.

## Supplementary Materials

The following supporting information can be downloaded at: https://www.mdpi.com/article/10.3390/jof10090604/s1. Figure S1: GO enrichment analysis of jointly upregulated genes; Figure S2: GO enrichment analysis of jointly downregulated genes; Figure S3: Heatmap of the DEPs involved in the peroxisome pathway; Table S1: The primer for qPCR used in this study. Table S2: Joint upregulation of expressed gene information. Table S3: Joint downregulation of expressed gene information,
