# Supplementary material for: Integrated Transcriptomics–Proteomics Analysis Reveals the Response Mechanism of Morchella sextelata to Pseudodiploöspora longispora Infection
Source: J Fungi (Basel). 2024 Aug 26;10(9):604. doi: 10.3390/jof10090604 (PMC11433447; doi:10.3390/jof10090604)
Supplement: Supplementary file 1 [file jof-10-00604-s001.zip › Supplementary figures.pdf]

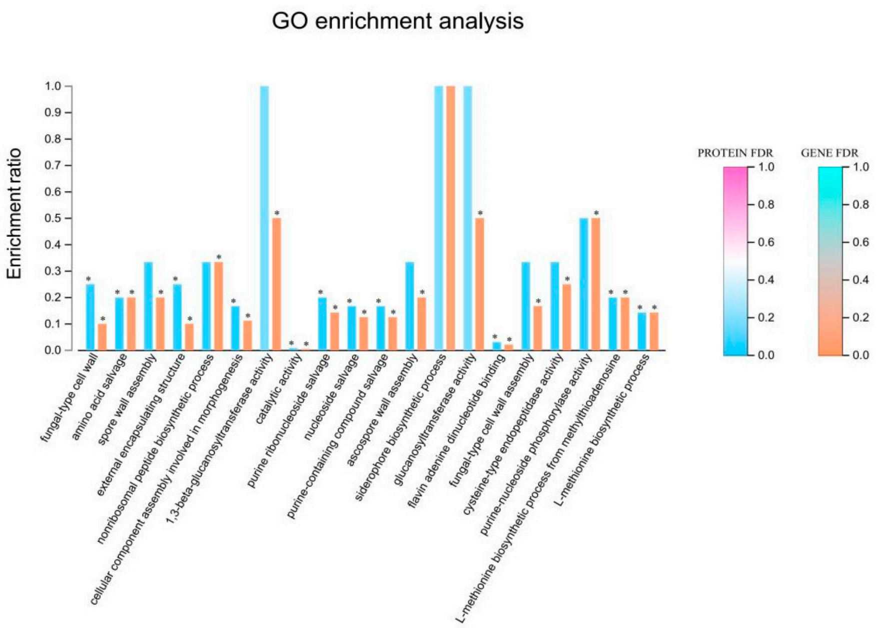

Figure S1 GO enrichment analysis of jointly upregulated genes.

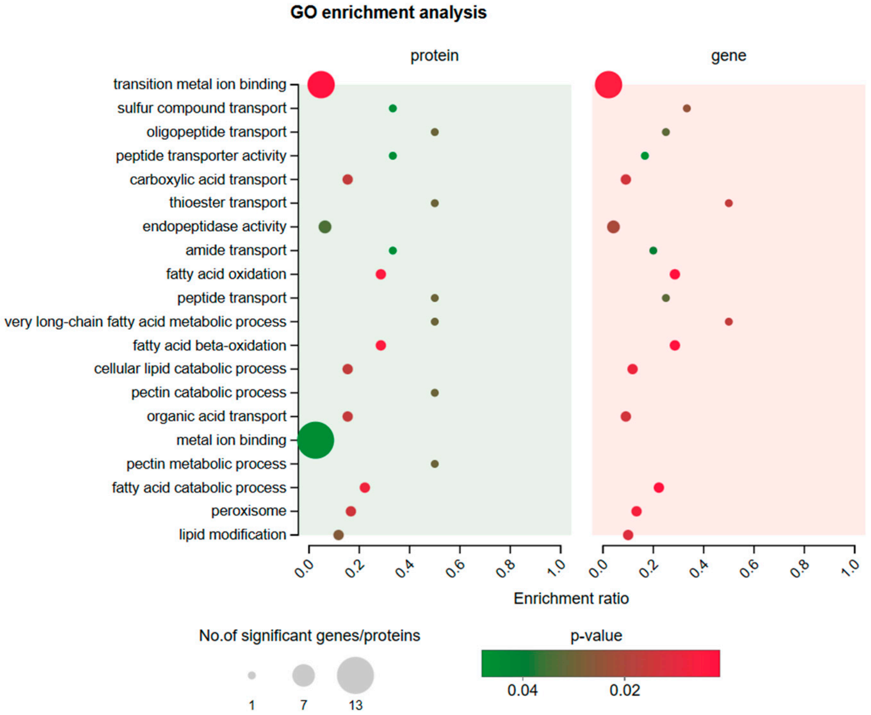

Figure S2 GO enrichment analysis of jointly downregulated genes.

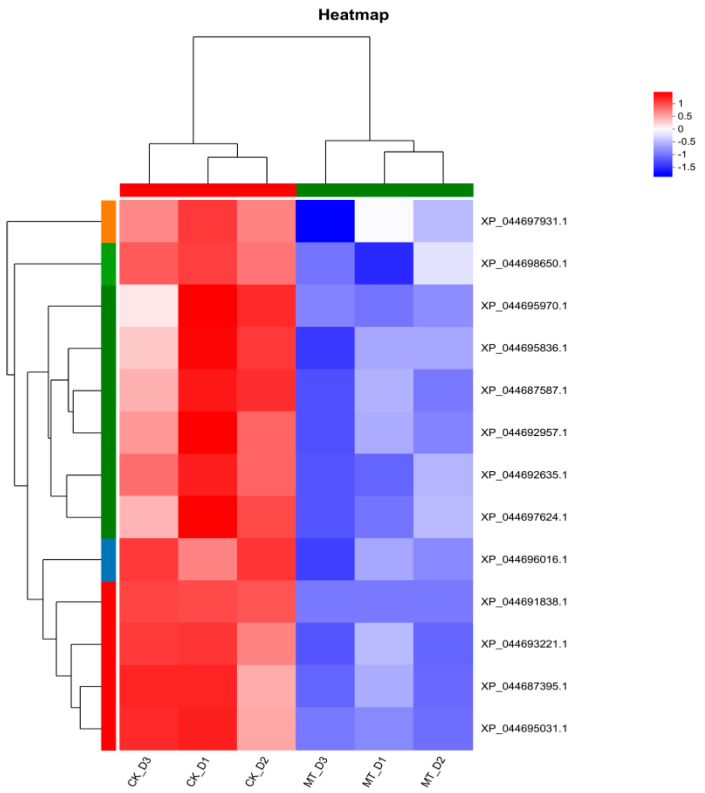

Figure S3 Heatmap of the DEPs involved in the peroxisome pathway.
