# Supplementary material for: Integrated Transcriptomics–Proteomics Analysis Reveals the Response Mechanism of Morchella sextelata to Pseudodiploöspora longispora Infection
Source: J Fungi (Basel). 2024 Aug 26;10(9):604. doi: 10.3390/jof10090604 (PMC11433447; doi:10.3390/jof10090604)
Supplement: Supplementary file 1 [file jof-10-00604-s001.zip › Table S1 The primer for qPCR used in this study.pdf]

gene id	Gene name	Name of the primer	Sequence (5' - 3')
gene-H6S33_012182	*PRX1*	g12182-F	CCGGTAAGAGCCCATATTCAC
		g12182-R	AATGCGGAAGTCAGGGAAG
gene-H6S33_004573	*SOD2*	g4573-F	GAGAGGTTTAAGGAAAGGTTTGG
		g4573-R	GTCTCAATCTCAAGCCTACCC
gene-H6S33_004442	*catB*	g4442-F	ATACTCAGCTCAACCGTAACG
		g4442-R	AAGGCTGTTTACGCTGTAGG
gene-H6S33_008738	*GAS4*	g8738-F	TCAACGACTCTAAGGGCAAAG
		g8738-R	GAGGAGGTAGAGGAGGACTTG
		g5983-F	CAACATTTGTCGAAGCGCC
gene-H6S33_005983	*FET3*	g5983-F	CAACATTTGTCGAAGCGCC
		g5983-R	ACTCCTATTCTCCCCACTGAG
gene-H6S33_000069	*ptr2*	g69-F	TCATCGTTTCAGCCCTTCC
		g69-R	TCGTTACTCTCATCCTGGTTTG
gene-H6S33_000010	*ORF9*	g10-F	CGATTTGGCTTTTGGTGATCC
		g10-R	GGGAATGAGTTGGCTGAGAG
gene-H6S33_005547	*PXMP2*	g5547-F	GCCCACTGAGAACAATGAAAAG
		g5547-R	GCCATTCCCGCAATAAACAG
gene-H6S33_006216	*Hxt1*	g6216-F	TTCTCCAGTTTCGATCCCAAG
		g6216-R	GGAGATACCGACAGACTTGAAG
Internal reference	5.8 S	5.8 S-F	CTCTTGGCTCTCGCATCGAT
		5.8 S-R	GGCGCAATGTGCGTTCA
